# Acute vision loss due to CML leukemic retinopathy reversed with leukapheresis

**DOI:** 10.1002/ccr3.7441

**Published:** 2023-06-02

**Authors:** Sally Leong, Tiffanie Do, Michael Shodiya, Jennifer Lee

**Affiliations:** ^1^ Division of Hematology and Medical Oncology, Department of Medicine Harbor‐UCLA Medical Center Torrance California USA; ^2^ Division of Hematology and Medical Oncology, Department of Medicine UCLA Ronald Reagan Medical Center Los Angeles California USA

**Keywords:** central retinal vein occlusion, chronic myeloid leukemia, cytoreduction, leukapheresis, retinopathy

## Abstract

Leukemic retinopathy is a severe complication of severe leukocytosis that results from untreated chronic myelogenous leukemia (CML). Immediate cytoreduction via leukapheresis may reverse ocular manifestations and prevent permanent vision damage. We present a case of a patient with acute unilateral vision loss found to have leukemic retinopathy in the setting of untreated CML with improvement of visual symptoms after leukapheresis and initiation of hydroxyurea.

## Graphical abstract

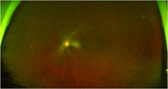

## 1 INTRODUCTION

Ophthalmologic manifestations associated with chronic myeloid leukemia are rare, but few prospective studies have shown that they are prevalent in 35–50% of all leukemic patients.^1^ They are most common in blast phase leukemias and less typically seen in chronic phase. Ocular involvement may precede the diagnosis or develop throughout the course of the disease. The exact mechanism of the involvement is unknown but may be due to the result of either primary infiltration of malignant cells or secondary to hematological changes and hemostasis from leukemia.

The most common manifestations of leukemic retinopathy include venous dilation and tortuosity. Retinal hemorrhages can also be seen during the development of this disease and are usually present in the posterior pole and accompanied by a white centre with leukemic cells, platelet‐fibrin aggregates or septic embolic. Cotton wool spots can also be seen. Hyperviscosity can also present as a bilateral central retinal vein occlusion.^2^ In cases of extreme leukocytosis (WBC >200,000 mm3), peripheral ischemia and neovascularisation can be seen.^1^ This last symptom is most notably seen in patients with chronic myeloid leukemia (CML). However, central retinal vein occlusions are rare symptomatic manifestations of CML. Patients may present with blurred vision or sudden, acute vision loss, even preceding typical symptoms of CML such as fever, weight loss, and fatigue.

The mainstay of treatment of leukemic retinopathy is chemotherapy targeted at the underlying disorder. However, leukapheresis is a treatment modality that has been shown in some case reports to have successfully reversed vision loss and ophthalmologic damage. Leukapheresis is emerging as an efficacious adjunct or initial treatment option for patients with severe retinopathy.

We present a case of severe leukemic retinopathy leading to acute unilateral vision loss that was successfully treated with leukapheresis along with hydroxyurea.

## 2 CASE PRESENTATION

**FIGURE 1:**
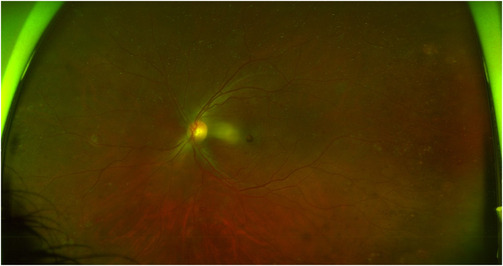
Colour ophthalmologic photo of the left eye showing numerous Roth spots in macula and periphery with intraretinal hemorrhages, cotton wool spots, and vascular sheathing concerning for central retinal vein occlusion.

A man in his early 40s with no past medical history presented to the emergency department with 3 days of left eye pain and associated worsening blurry vision. He was initially evaluated at a local optometry clinic and found to have retinal hemorrhages with decreased visual acuity and a central retinal vein occlusion (Figure 1) and referred to the emergency department. At the time of initial presentation, he complained of a white spot that obscured his vision. He also endorsed loss of appetite for 1 week without weight loss. He denied fevers, chills, night sweats or a personal or family history of cancer. He was hemodynamically stable. Pertinent physical exam findings were hepatomegaly and splenomegaly.

Initial laboratory values were significant for leukocytosis of 477 K/cumm with 10% blasts, 4% basophils, 4% eosinophils, and platelets of 276 K/cumm. Computed tomography imaging was notable for hepatomegaly and a markedly enlarged spleen with mass effect on the organs of the left hemi‐abdomen. Bone marrow biopsy was performed with results indicating marked leukocytosis and circulating blasts of 7%. BCR‐ABL genetic test was obtained and was positive, confirming the diagnosis of CML.

A femoral central venous catheter was placed and the patient was initiated on leukapheresis on day one of admission. He was given 3 g of hydroxyurea in the emergency department and then started on hydroxyurea 2 g twice a day. White blood count initially increased to 533 K/cumm and 487 K/cumm after leukapheresis. Over the course of his hospitalisation, his visual symptoms improved after leukapheresis with nearly total improvement of his left visual deficits within 2 days. His leukocytosis began to resolve in the days following leukapheresis, initially decreasing to 340 K/cumm the day after the procedure and decreased to 245 K/cumm on hospital day four.

The patient was discharged on hospital day 4 with 2 grams of hydroxyurea three times a day for 2 weeks with planned hematology follow‐up and initiation of a tyrosine kinase inhibitor.

**FIGURE 2:**
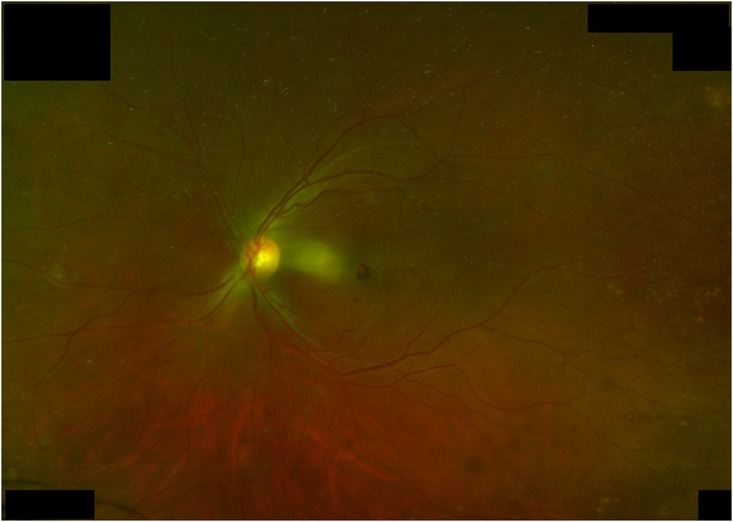
Colour ophthalmologic photo of the left eye post‐leukapheresis showing improving intraretinal hemorrhages, mild cotton wool spots in the periphery, improving vascular sheathing, and new intraretinal hemorrhages in the macula.

Two weeks after, ophthalmologic photos were obtained demonstrating improvement of the central retinal vein occlusion, intraretinal hemorrhages, and cotton wool spots (Figure 2). At follow‐up 2 years after initial presentation, the patient remains on maintenance dasatinib with no permanent vision changes.

## 3 DISCUSSION

Ocular complications may manifest as a symptom of severe, untreated leukemia. Although the pathophysiology behind ocular manifestations of CML is not well understood, it is most likely multifactorial. These mechanisms likely include infiltration by neoplastic cells leading to reduced blood flow, vascular stagnation, ischemia, and anemia.^1^, ^3^ There may also be thrombosis due to toxic products released by leukemic cells, and increased amounts of angiogenic factors that lead to elevated levels of vascular endothelial growth factor, fibroblast growth factor 2, and matrix metalloproteinases.^3^ Severe, symptomatic leukocytosis is associated with an extremely high mortality rate that can lead to both respiratory failure and permanent neurological complications. When signs of organ failure begin to present, the death rate at 1 week approaches 90 %.^4^ Thus, prompt cytoreduction is necessary to improve clinical outcomes.

Although the mainstay of treatment for leukemic retinopathy remains induction chemotherapy at the underlying disorder, leukapheresis has become more common as an adjunctive or initial treatment. Prompt, effective reduction of the high white cell count can cause rapid improvement of visual symptoms. Leukapheresis aids in prompt reversal of microvascular sludging and should be considered for rapid cytoreduction during the initial stages, since drugs such as hydroxyurea or BCR‐ABL tyrosine kinase inhibitors take a longer time to demonstrate their cytoreductive effects.^5^, ^6^ The process of leukapheresis involves removing whole blood from a patient through a central venous catheter, separating leukocytes out through an apparatus, and then transfusing the cytoreduced blood back to the patient. It is an efficacious procedure to rapidly reduce leukocytosis without necessitating donor transfusions or additional medications.

However, leukapheresis remains controversial as it has variable results in overall mortality benefits and has not been well‐studied in different types of leukemia. Larger prospective studies have not shown consistent benefits or decreases in early morality rates and have only been studied in acute myeloid leukemia (AML) cases.^5^, ^7^, ^8^ One study showed that although patients had a significant reduction in leukocytosis after therapeutic leukapheresis, they also displayed a higher death rate within the first 24 hours and an overall higher early death rate compared to patients with chemotherapy alone.^9^ However, this study only looked at patients with AML rather than CML, although acute blast phase CML is treated similarly. In the 2021 review article by Yassin, et al, ophthalmologic manifestations from CML could only be found in the literature as case reports or case series.^2^ Of the 38 CML cases reviewed, only two were treated with emergent leukapheresis; both cases showed little change in outcome.^2^, ^10^, ^11^ This differs from our case in that significant improvement in vision was seen in 2 days. Leukapheresis for acute, symptomatic hyperleukocytosis currently is a grade 2B recommendation based on the American Society of Apheresis (ASFA), with most of its studies derived from AML cases as leukemic complications are more often seen in AML rather than CML.^12^ Grade 2B recommendation is defined as a weak recommendation with moderate‐quality evidence as per the ASFA. This likely stems from the variable outcomes seen in the reported AML cases that have been treated with leukapheresis, the paucity in literature of leukemic complications of CML treated with leukapheresis, and no improvement in outcome or even an association with early mortality in APL cases.^12^ Lastly, its generalisability is limited in its accessibility as leukapheresis is an invasive procedure that requires a large bore central venous catheter and is only offered in select medical centers. Despite these limitations, given the few amount of other initial existing treatments to rapidly reduce leukocytosis, it may be a beneficial therapy to treat acute, severe consequences of extreme leukocytosis. In our patient, early initiation of leukapheresis, along with hydroxyurea therapy, resulted in reversal of leukemia‐associated retinal changes.

Although leukapheresis in current studies have not been proven to have overall mortality benefits, our case shows that there may be a role for this procedure in reducing permanent vascular damage. Our patient successfully had reversal of his acute vision loss and was able to initiate chemotherapy shortly after his initial presentation. Further studies are needed to demonstrate the benefits of symptom relief of severe leukocytosis in conjunction with chemotherapy and hydroxyurea.

## AUTHOR CONTRIBUTIONS

**Sally Leong:** Data curation; investigation; writing – original draft; writing – review and editing. **Tiffanie Do:** Data curation; writing – review and editing. **Michael Shodiya:** Conceptualization; writing – review and editing. **Jennifer Lee:** Conceptualization; supervision; writing – review and editing.

## FUNDING INFORMATION

There was no funding obtained for this article. This article was written under the employment of the above affiliations.

## CONFLICT OF INTERESTS STATEMENT

SL, TD, MS, JL have no conflicts of interest to declare.

## CONSENT

Written informed consent was obtained from the patient to publish this report in accordance with the journal's patient consent policy.

## Data Availability

The data that support the findings of this study are available on request from the corresponding author. The data are not publicly available due to privacy or ethical restrictions.
